# Supplementary material for: Assessment of Diversity of Culturable Marine Yeasts Associated with Corals and Zoanthids in the Gulf of Thailand, South China Sea
Source: Microorganisms. 2020 Mar 26;8(4):474. doi: 10.3390/microorganisms8040474 (PMC7232451; doi:10.3390/microorganisms8040474)
Supplement: Supplementary file 1 [file microorganisms-08-00474-s001.pdf]

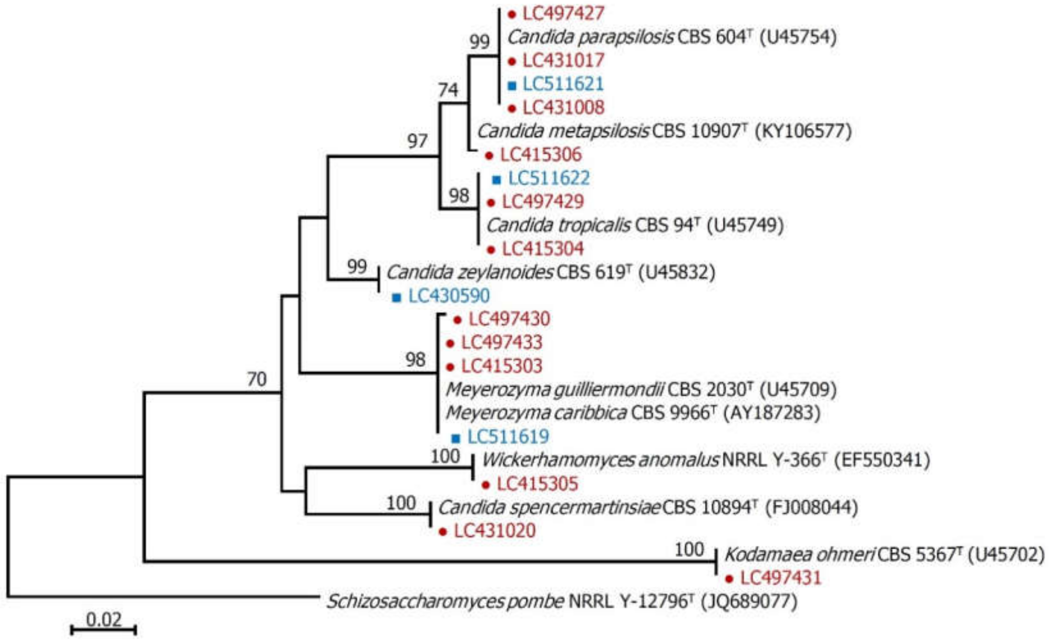

Figure S1. Phylogenetic relationship based on the sequence of the D1/D2 domain of the LSU rRNA gene of yeast species isolated from corals and zoanthids in sampling site S1 (●) and sampling site S2 (■) in the phylum Ascomycota, and their closely related yeast species retrieved from the GenBank databases. The tree was constructed with the maximum-likelihood method and the general time reversible (GTR) evolutionary model in the MEGA software version 7.0. Numbers at nodes indicate the bootstrap percentage (>50%) derived from 1000 datasets. *Schizosaccharomyces pombe* NRRL Y-12796^{T} (JQ689077) was used as the outgroup. The scale bar indicates an evolutionary distance of 0.02 K_{nuc}.

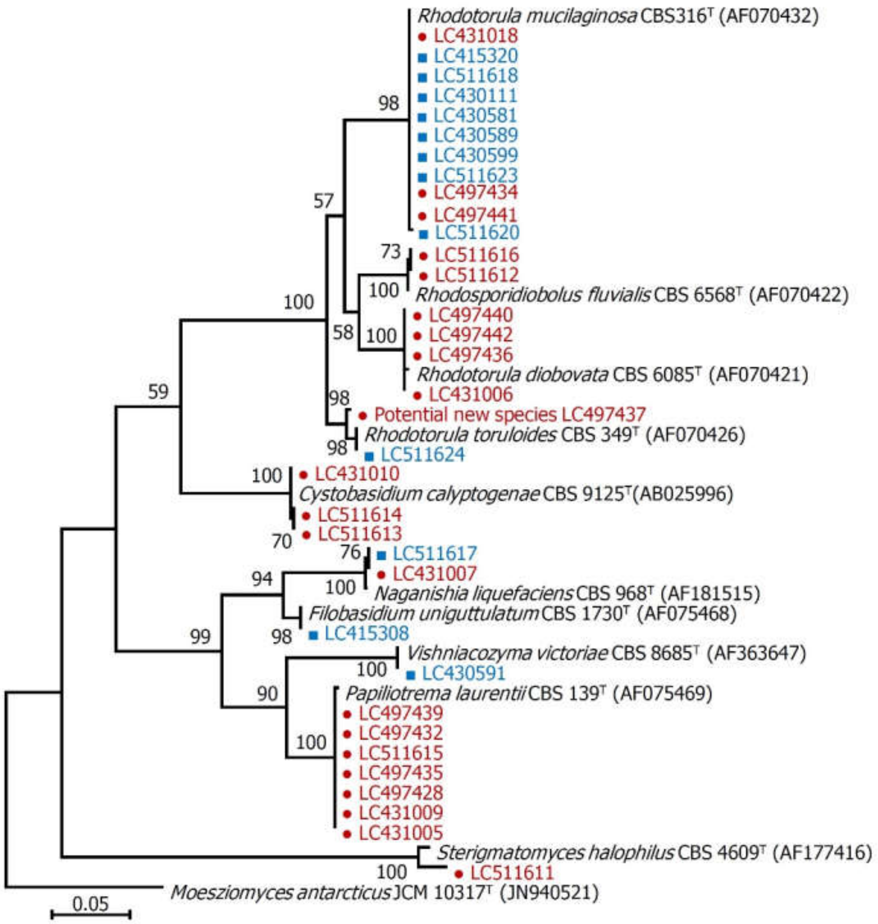

Figure S2. Phylogenetic relationship based on the sequence of the D1/D2 domain of the LSU rRNA gene of yeast species isolated from corals and zoanthids in sampling site S1 (●) and sampling site S2 (■) in the phylum Basidiomycota, and their closely related yeast species retrieved from the GenBank databases. The tree was constructed with the maximum-likelihood method and the GTR evolutionary model in the MEGA software version 7.0. Numbers at nodes indicate the bootstrap percentage (>50%) derived from 1000 datasets. *Moesziomyces antarcticus* JCM 10317^T (JN940521) was used as the outgroup. The scale bar indicates an evolutionary distance of 0.05 K_{nuc}.

Table S1. Identification of yeast isolated from corals and zoanthids by analysis of the D1/D2 domain of the LSU rRNA gene and the ITS region.

Strain DMKU	Closest species (Accession no. of D1/D2; ITS)	D1/D2 domain				ITS region				Result of identification
		Accession no.	No. of identity	No. of gap	No. of substitution	Accession no.	No. of identity	No. of gap	No. of substitution	
**Coral samples**										
J7-1	*Papiliotrema laurentii* CBS139^{T} (AF075469)	LC431005	573/574	0	1	-	-	-	-	*P. laurentii*
JC7-2	*Rhodotorula diobovata* CBS6085^{T} (AF070421)	LC431006	532/533	0	1	-	-	-	-	*R. diobovata*
JC7-4	*Naganishia liquefaciens* CBS968^{T} (AF181515)	LC431007	555/556	0	1	-	-	-	-	*N. liquefaciens*
JC7-5	*Candida parapsilosis* CBS604^{T} (U45754)	LC431008	528/528	0	0	-	-	-	-	*C. parapsilosis*
JC8-1	*Papiliotrema laurentii* CBS139^{T} (AF075469)	LC431009	571/572	0	1	-	-	-	-	*P. laurentii*
JC9-1	*Cystobasidium calyptogenae* CBS9125^{T} (AB025996)	LC431010	565/566	0	1	-	-	-	-	*Cys. calyptogenae*
JC14-1	*Candida parapsilosis* CBS604^{T} (U45754)	LC431017	519/519	0	0	-	-	-	-	*C. parapsilosis*
JC15-1	*Rhodotorula mucilaginosa* CBS316^{T} (AF070432)	LC431018	543/544	0	1	-	-	-	-	*R. mucilaginosa*
J18-1	*Candida spencermartinsiae* CBS10894^{T} (FJ008044)	LC431020	505/505	0	0	-	-	-	-	*C. spencermartinsiae*
JC22-1	*Candida parapsilosis* CBS604^{T} (U45754)	LC497427	535/535	0	0	-	-	-	-	*C. parapsilosis*
JC24-1	*Sterigmatomyces halophilus* CBS4609^{T} (AF177416)	LC511611	585/592	6	1	-	-	-	-	*S. halophilus*
JC25-1	*Papiliotrema laurentii* CBS139^{T} (AF075469)	LC497428	606/607	0	1	-	-	-	-	*P. laurentii*
JC25-2	*Candida tropicalis* CBS94^{T} (U45749)	LC497429	529/529	0	0	-	-	-	-	*C. tropicalis*
J33-2	*Rhodotorula mucilaginosa* CBS316^{T} (AF070432)	LC497434	532/533	0	1	-	-	-	-	*R. mucilaginosa*
J33-3	*Papiliotrema laurentii* CBS139^{T} (AF075469)	LC497435	579/580	0	1	-	-	-	-	*P. laurentii*
JC33-1	*Rhodotorula diobovata* CBS6085^{T} (AF070421)	LC497436	549/550	0	1	-	-	-	-	*R. diobovata*
JC33-2	*Rhodosporidiobolus fluvialis* CBS6568^{T} (AF070422; NR077089)	LC511612	535/538	0	3	LC522501	593/598	0	5	*Rh. fluvialis*
JC33-4	*Cystobasidium calyptogenae* CBS9125^{T} (AB025996; AB025996)	LC511613	607/609	0	2	LC522502	557/562	1	4	*Cys. calyptogenae*
JC35-1	*Rhodotorula diobovata* CBS6085^{T} (AF070421)	LC497442	532/533	0	1	-	-	-	-	*R. diobovata*

Strain DMKU	Closest species (Accession no. of D1/D2; ITS)	D1/D2 domain				ITS region				Result of identification
		Accession no.	No. of identity	No. of gap	No. of substitution	Accession no.	No. of identity	No. of gap	No. of substitution	
JC35-2	*Cystobasidium calyptogenae* CBS9125^T (AB025996; AB025996)	LC511614	607/609	0	2	LC522503	559/564	1	4	*Cys. calyptogenae*
JC46-1	*Papiliotrema laurentii* CBS139^T (AF075469)	LC511615	562/563	0	1	-	-	-	-	*P. laurentii*
C322-1	*Filobasidium uniguttulatum* CBS1730^T (AF075468)	LC415308	564/564	0	0	-	-	-	-	*F. uniguttulatum*
3222-1	*Rhodotorula mucilaginosa* CBS316^T (AF070432)	LC415320	552/555	2	1	-	-	-	-	*R. mucilaginosa*
3222-2	*Naganishia liquefaciens* CBS968^T (AF181515)	LC511617	596/597	0	1	-	-	-	-	*N. liquefaciens*
3236-1	*Rhodotorula mucilaginosa* CBS316^T (AF070432)	LC511618	554/555	0	1	-	-	-	-	*R. mucilaginosa*
729-1	*Rhodotorula mucilaginosa* CBS316^T (AF070432)	LC430111	554/555	1	0	-	-	-	-	*R. mucilaginosa*
7231-1	*Rhodotorula mucilaginosa* CBS316^T (AF070432)	LC430581	550/550	0	0	-	-	-	-	*R. mucilaginosa*
7235-1	*Rhodotorula mucilaginosa* CBS316^T (AF070432)	LC430589	549/550	0	1	-	-	-	-	*R.mucilaginosa*
C7235-3	*Candida zeylanoides* CBS619^T (U45832)	LC430590	515/515	0	0	-	-	-	-	*C. zeylanoides*
C7235-4	*Vishniacozyma victoriae* CBS8685^T (AF363647)	LC430591	550/550	0	0	-	-	-	-	*V. victoriae*
7241-1	*Rhodotorula mucilaginosa* CBS316^T (AF070432)	LC430599	555/556	1	0	-	-	-	-	*R. mucilaginosa*
FC12-1	*Meyerozyma caribbica* CBS9966^T (AY187283)	LC511619	519/519	0	0	-	-	-	-	*M. caribbica*
FC12-2	*Rhodotorula mucilaginosa* CBS316^T (AF070432)	LC511620	533/534	0	1	-	-	-	-	*R. mucilaginosa*
FC12-3	*Candida parapsilosis* CBS604^T (U45754)	LC511621	524/524	0	0	-	-	-	-	*C. parapsilosis*
FC12-4	*Candida tropicalis* CBS94^T (U45749)	LC511622	523/523	0	0	-	-	-	-	*C. tropicalis*
J37-1	*Rhodotorula toruloides* CBS349^T (AF070426; AF444489)	LC497437	583/588	0	5	LC522504	579/588	3	6	Potential new species closest to *R. toruloides*
**Zoanthid samples**										
319-1	*Meyerozyma guilliermondii* CBS2030^T (U45709)	LC415303	542/545	1	2	-	-	-	-	*M. guilliermondii*
319-2	*Candida tropicalis* CBS94^T (U45749)	LC415304	544/545	1	0	-	-	-	-	*C. tropicalis*

Strain DMKU	Closest species (Accession no. of D1/D2; ITS)	D1/D2 domain				ITS region				Result of identification
		Accession no.	No. of identity	No. of gap	No. of substitution	Accession no.	No. of identity	No. of gap	No. of substitution	
C319-1	*Wickerhamomyces anomalus* NRRL_Y-366^{T} (EF550341)	LC415305	553/556	2	1	-	-	-	-	*W. anomalus*
C319-2	*Candida metapsilosis* CBS10907^{T} (KY106577)	LC415306	552/554	1	1	-	-	-	-	*C. metapsilosis*
J29-5	*Meyerozyma guilliermondii* CBS2030^{T} (U45709)	LC497430	532/534	0	2	-	-	-	-	*M. guilliermondii*
JC29-2	*Kodamaea ohmeri* CBS5367^{T} (U45702)	LC497431	442/442	0	0	-	-	-	-	*K. ohmeri*
JC29-6	*Papiliotrema laurentii* CBS139^{T} (AF075469)	LC497432	573/574	0	1	-	-	-	-	*P. laurentii*
JC29-7	*Meyerozyma caribbica* CBS9966^{T} (AY187283)	LC497433	519/519	0	0	-	-	-	-	*M. caribbica*
JC34-1	*Papiliotrema laurentii* CBS139^{T} (AF075469)	LC497439	576/577	0	1	-	-	-	-	*P. laurentii*
JC34-2	*Rhodotorula diobovata* CBS6085^{T} (AF070421)	LC497440	532/533	0	1	-	-	-	-	*R. diobovata*
JC34-5	*Rhodosporidiobolus fluvialis* CBS6568^{T} (AF070422; NR077089)	LC511616	546/549	0	3	LC522505	588/593	0	5	*Rh. fluvialis*
JC34-7	*Rhodotorula mucilaginosa* CBS316^{T} (AF070432)	LC497441	533/534	0	1	-	-	-	-	*R. mucilaginosa*
F9-1	*Rhodotorula mucilaginosa* CBS316^{T} (AF070432)	LC511623	533/534	0	1	-	-	-	-	*R. mucilaginosa*
FC9-1	*Rhodotorula toruloides* CBS349^{T} (AF070426)	LC511624	532/532	0	0	-	-	-	-	*R. toruloides*
